# Concertation of Anti-Reflective, Superhydrophobic Surface Based on Rational Assembly of Dual-Size Silica

**DOI:** 10.3390/ma18245601

**Published:** 2025-12-12

**Authors:** Lu Xu, Lei Niu, Shuqun Chen, Ting He, Junshu Wu, Jianbo Ai, Yongli Li

**Affiliations:** 1 China Helicopter Research and Development Institute, Jingdezhen 333001, China; 2 State Key Laboratory of Materials Low-Carbon Recycling, College of Materials Science and Engineering, Beijing University of Technology, Beijing 100124, China

**Keywords:** anti-reflective superhydrophobic surfaces, particle gradation, dual-size assembly, robustness

## Abstract

Silica-based multifunctional coatings hold great promise for applications in optical devices, lenses, and solar panels. Herein, we report a facile, low-temperature route to integrate super-hydrophobicity with high transparency and low haze. By precisely controlling particle gradation and applying fluorine passivation, a multi-scale structure with micro-scale uniformity and nano-scale asperity was constructed. This unique architecture, combined with low surface energy, effectively reduces light scattering and enhances air trapping. Consequently, the coated glass achieves a high optical transmittance of 95.24% with a low haze of 0.97%, alongside a water contact angle of 153° and a sliding angle of 3°. The coating also exhibits distinct anti-reflection (an improvement of ~5.0% relative to the bare substrate) and self-cleaning properties. Furthermore, it demonstrates impressive robustness and durability, withstanding extreme conditions including cryogenic temperatures (−50 °C), hygrothermal environments, and long-term outdoor exposure. This work demonstrates the versatile potential of our strategy for fabricating highly transparent and superhydrophobic surfaces.

## 1. Introduction

Transparent hydrophobic surfaces hold significant potential for applications in various fields, including optical windows [1,2,3], display devices [4,5,6], solar panels [7,8,9,10] and medical equipment [11,12,13,14]. These surfaces exhibit strong resistance to the adhesion of moisture and other polar liquids while maintaining high optical transparency across the visible spectrum (380–780 nm). However, an inherent trade-off exists between hydrophobicity and optical transparency. Inspired by the lotus leaf structure, achieving super-hydrophobicity—typically defined by a static contact angle greater than 150° and a sliding angle below 10°—requires high surface roughness and low surface energy [15,16,17]. In contrast, optical transparency relies on the use of low-refractive-index materials, a flat surface morphology, and an appropriate coating thickness. The wetting behavior of such surfaces can be described by the Cassie–Baxter non-wetting model [18].

(1) $$\cos\theta_{r}^{c}=f\left(\cos\theta_{0}+1\right)-1\;$$

where $\theta_{r}^{c}$ is the Cassie–Baxter contact angle, *f* is the fraction of liquid–solid contact (*f* < 1), and *θ*_0_ is Young’s contact angle. Therefore, achieving high hydrophobicity relies on both low surface energy (*θ*_0_ > 90°) and minimized solid–liquid contact area—typically realized through a highly rough surface texture—which promotes the formation of air pockets between water and the substrate and prevents water droplets from pinning on the surface. Numerous studies have demonstrated that super-hydrophobicity can be attained by fabricating microscale ridges and grooves on various substrates [19,20,21]. However, excessive surface roughness enhances light scattering and leads to a pronounced reduction in optical transmittance [22,23]. According to Rayleigh scattering, when the size of the surface roughness is less than the wavelength of the incident light, the intensity of scattered light, *I*, is

(2) $$\frac{I}{I_{0}}=\frac{1+{cos}^{2}\theta }{2S^{2}}{\left(\frac{2\pi }{\lambda }\right)}^{4}{\left(\frac{n^{2}-1}{n^{2}+2}\right)}^{2}{\left(\frac{d}{2}\right)}^{6}$$

where *I*_0_ is the intensity of incident light, *S* is the distance between the surface and the detector, *n* is the refractive index of the particle, *d* is the particle size and *λ* is wavelength.

The intensity of light scattering depends on both surface roughness and the refractive index of the coating material, suggesting a viable pathway to concurrently achieve high transparency and hydrophobicity. To minimize Rayleigh scattering, one strategy is to reduce the effective refractive index of the coating [24]. For example, Li et al. [25] fabricated a nanoscale porous structure using hydrophobic fumed silica nanoparticles, which lowered the effective refractive index by 23% compared to that of solid silica. The resulting coating exhibited both excellent transparency and super-hydrophobicity, even at relatively low roughness. Besides constructing porous microstructures, hollow silica nanospheres can also be employed to decrease the effective refractive index [26]. Another approach involves restricting the size of scattering features to below one-tenth of the wavelength of visible light, typically under 100 nm. Following this principle, a key factor in combining super-hydrophobicity with high optical transparency is to create a hierarchical rough structure comprising multi-scale grooves while maintaining low overall roughness [27].

It is widely acknowledged that dual-size gradation is superior to single-size gradation in the fabrication of superhydrophobic transparent coatings [28,29,30]. Under the dual-size particles, the larger particles act as the skeleton to construct the rough surface, and the smaller ones occupy the gaps. Karunakaran et al. [28] obtained a superhydrophobic silica coating with a water contact angle (WCA) of 157° on the surface of Si wafer by sequentially impregnating two different size SiO_2_, 20 and 100 nm, respectively. When applied to a glass substrate, the optical transmittance reached almost 100% of the substrate. Lee et al. [29] obtained a superhydrophobic coating with an optical transmittance greater than 90% (WCA is higher than 160°) by adjusting the proportion of dual-size nanoparticles. Zhao et al. [30] reported a transparent superhydrophobic coating by uniformly coating hydrophobic/hydrophilic dual-size SiO_2_ particles separately. Liu et al. [31] prepared a transparent coating with a WCA of 164° by post-calcination of the PDMS/SiO_2_ coating. Indeed, this assembled structure possesses advantages: (1) it helps to control the particle accumulation and avoid excessive fluctuation at micro- and larger scale, which lowers the scattering size; (2) it enables the formation of sub-rough structures at nano-scale, and adjust the groove size distribution, reducing the liquid–solid interface fraction. Although current studies have found a method to balance super-hydrophobicity and transparency, there is a certain degree of loss in the optical transmittance; actually, few studies have paid attention to haze of the coating, apart from transparency. As an important parameter of transparency, haze describes the portion of light scattered at an angle of larger than 2.5° from the incident light when passing through a transparent object, which has a significant influence on imaging precision [32,33]. High haze is not suitable for applications such as flexible displays and tough screens, which require high transparency and clarity [34]. Consequently, a pivotal challenge is the tight control of Rayleigh scattering for low haze, as this fundamentally requires striking a precise balance between transparency and super-hydrophobicity. Their simultaneous optimization continues to pose a major research challenge. The influence of precise proportion and size gradation on coating structure and resultant performance is also lacking. Notably, dual-size assembled SiO_2_ coating can be prepared by sol-gel derived silica nanoparticles followed by dip coating [35], spin coating [36], and spray coating [37]. Among these methods, dip-coating offers an attractive approach for thin-film fabrication on varied surface geometries, given its process maturity, scalability, flexibility, and cost-effectiveness [38].

In this work, a highly transparent hydrophobic surface was fabricated on glass substrate using sol-gel derived dual-size silica nanoparticles followed by low temperature (≤70 °C) dip coating and passivation treatment. By controlling the hydrolysis of TEOS, a series of SiO_2_ sols with different particle sizes were first synthesized. The dual-size assembly, governed by a sphere dense packing model, allowed the coating properties to be optimized, resulting in enhanced optical transmittance, ultra-low haze, and super-hydrophobicity when compared to bare glass. The coatings also exhibited remarkable resistance to low temperatures (–50 °C) and hygrothermal conditions (60 °C, 95% RH). Furthermore, the versatility of the coating was demonstrated by forming transparent hydrophobic surfaces on polymethyl methacrylate (PMMA), cloth, steel, and wood.

## 2. Materials and Methods

### 2.1. Materials

All chemicals were used as received, including tetraethyl orthosilicate (TEOS) (98%, Macklin, Inc., Shanghai, China), ammonium hydroxide (25–28%, China National Medicines Co., Ltd., Beijing, China), anhydrous ethanol (99%, China National Medicines Co., Ltd., Beijing, China), hydrochloric acid (37%, Beijing Chemicals Co., Ltd., Beijing, China), (heptadecafluoro-1,1,2,2-tetradecyl) trimethoxy-silane (FAS-17) (99%, Gelest, Inc., Morrisville, PA, USA).

### 2.2. Synthesis of Silica Sol with Different Nanoparticle Size (NPs)

Silica sol was synthesized by base-induced Stöber chemistry. Briefly, 60 mL of ethanol, 2 mL of pure water and 2 mL of ammonium hydroxide were mixed, followed by adding 30 mL of ethanol containing 6 mL of TEOS. The solution mixture was then held at 55 °C in a water bath for 12 h. Prior to use, the as-synthesized silica sol was further heated at the same temperature in a ventilating cabinet for at least 6 h to volatilize NH_3_ until the total volume dropped to about 60 mL and then ethanol was supplemented to a total volume of 100 mL. By the increase in water addition to accelerate the hydrolysis of TEOS, ca. 0.5, 2.0, 6.0, and 8.0 mL, SiO_2_ NPs with average sizes of 20, 50, 70 and 90 nm were synthesized.

### 2.3. Synthesis of Semi-Hydrolyzed Silica Sol

Semi-hydrolyzed silica (SHS) sol was synthesized according to a similar procedure using only hydrochloric acid as the catalyst. In a typical synthesis, a solution that consists of 30 mL of ethanol and 6 mL of TEOS was poured into a mixture of 40 mL of ethanol, 24 mL of pure water and 0.5 mL of hydrochloric acid, then was kept at 50 °C in a water bath for 6 h.

### 2.4. Preparation of Dual-Scale Assembled Silica Coatings

In all preparations, we fixed one size as 20 nm SiO_2_ NPs and separately changed the size of the other, i.e., 50, 70, 90 nm, to constitute the particle gradation. The dual-size NP sol was well-mixed with SHS sol to form a precursor of the coating. To prepare coatings, the washed and dried substrate was immersed in composite sol for 60 s and withdrawn at a rate of 1200 μm s^−1^ on dip-coating equipment. This was repeated twice with a 10 min interval. The obtained coating was dried, and subsequently placed in a desiccator containing 50 μL of FAS-17 in a separate petri dish for vapor deposition at 70 °C in an oven for 4 h. The as-prepared coating was denoted as S_20/X_, where subscript X represents the selected size of SiO_2_ NPs, e.g., S_20/70_ denotes that the precursor is composed of 20 and 70 nm SiO_2_ NPs. As a control group, single-size coatings were prepared according to the same procedure.

### 2.5. Characterizations

Optical transparency was evaluated using a UV-vis 3600 spectrophotometer (Shimadzu, Kyoto, Japan) equipped with a BaSO_4_ integrating sphere. The average light transmittance (380–780 nm) is calculated based on the international standard ISO 13468-2 [39].

(3) $$T_{T}=\frac{\sum_{\lambda =380}^{780}S\left(\lambda \right)\times T_{T}\left(\lambda \right)\times V\left(\lambda \right)}{\sum_{\lambda =380}^{780}S\left(\lambda \right)\times V\left(\lambda \right)}$$

where T_T_ is total transmittance of D65 standard light source, S(λ) is the relative spectral power distribution of the light source and V(λ) is the spectral luminous efficiency.

Haze (H) was obtained according to the British standard EN 673 [40], which was measured on the same UV-vis spectrophotometer.

Static water contact angle was determined on a SZ-CAMB1 instrument (Shanghai Sunzern Instrument Co., Ltd. Shanghai, China) from a 50 μL water droplet. For water sliding angle (WSA) measurement, the sample was placed on a custom-designed stage with a protractor attached to it, and a 50 μL water droplet was used. The contact angle and sliding angle were obtained by measuring at least three different positions on each sample.

Fourier transform infrared (FTIR) spectrum of the sample was obtained at PerkinElmer Spectrum 100 using the KBr compression method.

X-ray photoelectron spectroscopy (XPS) was performed on an ESCALAB 250Xi (Thermo Fisher Scientific, Waltham, MA, USA), in which Al Kα (hν = 1487 eV) was used as the excitation light source, the operating voltage was set at 15 kV, the test power was 300 W, and the test results were corrected with standard carbon C1s (284.8 eV).

Topography of the coating was obtained using a multimodal atomic force microscope (AFM, Bruker Dimension Icon, Santa Barbara, CA, USA) with a Si_3_N_4_ cantilever in tapping mode. The thickness and roughness of samples were measured according to the AFM scan.

The surface morphology of the samples was imaged by field emission scanning electron microscopy (FESEM, HITACHI S-4800, Tokyo, Japan) with acceleration potential of 15 kV.

Transmission electron microscopy (TEM) observation was conducted on a Thermo Scientific F200X (Waltham, MA, USA), and elemental composition was measured with an energy spectrum analyzer attached to the TEM.

A 3M transparent tape was applied to the tape peeling test. The test was repeated and the related properties were recorded after every two peelings.

In the low-temperature tolerance test, the sample was kept in a CZ-A-100G low-temperature test chamber at −50 °C for 7 and 14 days, respectively, and then allowed the temperature up to 20 °C. Damp heat test was performed in a STH-100 aging test chamber with an operating temperature of 60 °C and a relative humidity of 95% for 10 days overall. After each test, the optical transmittance, haze and WCA were separately evaluated.

## 3. Results

### 3.1. Structure and Components of the Prepared Coatings

**Figure 1:**
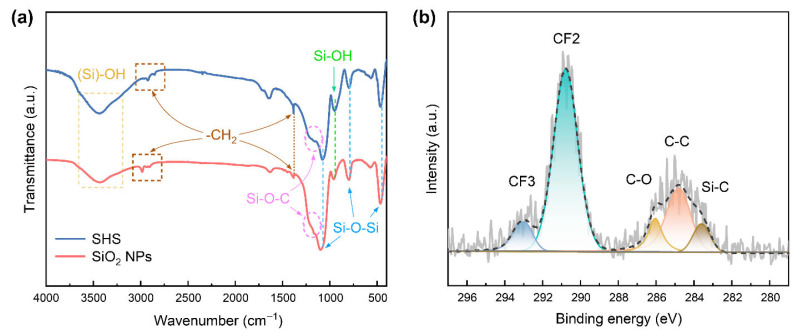
(**a**) FTIR spectra of acid/base-induced SiO_2_ and (**b**) C1s XPS spectrum of fluorinated coating.

By carefully controlling the synthesis conditions, a series of SiO_2_ nanoparticles with predefined sizes was fabricated (Figure S1). The typical FTIR spectra of the as-synthesized SiO_2_ NPs and SHS show that the broad absorption band centered at 3433 cm^−1^ is attributed to the stretch of -OH groups generated by Si-OH band motion, the band at 949 cm^−1^ arises from the Si-OH vibration, and bands at 1094, 796 and 461 cm^−1^ are assigned to Si-O-Si vibrations (Figure 1a) [41]. Compared with SiO_2_ prepared via base catalysis, SHS displays more distinct vibration signals corresponding to Si–OH and –CH_2_ groups, while the Si–O–Si signal is relatively weaker. This can be attributed to incomplete hydrolysis under weakly acidic conditions, which results in hydroxyl-rich surfaces on the colloidal clusters. These abundant –OH groups may serve as hydrogen-bond acceptors and donors, thereby promoting strong inter-cluster interactions through hydrogen bonding [42]. Thus, the observed gel-like network morphology of the SHS can be understood in this context (Figure S2). It can be further inferred that the incorporation of SHS improves coating–substrate adhesion via chemical bonding, thereby enhancing mechanical robustness. The reduction of Si-OH and Si-O-C vibration intensity with increasing particle size for base-induced SiO_2_ was also observed (Figure S3), which is a result of further development of hydrolysis and condensation. The XPS survey shows that the coating surface before passivation is composed of Si and O elements. Beyond that, the passivated surface presents a strong peak from the F element (Figure S4); moreover, from the high-resolution C1s spectrum, binding energies at 283.6, 284.8, 286.1, 290.1 and 293.1 eV correspond to Si-C, C-C, C-O, -CF_2_ and -CF_3_, respectively (Figure 1b) [43]. This result confirms that F-grafted SiO_2_ coatings were produced by an ordinary vapor deposition.

### 3.2. Optical Properties and Wettability of the Single-Size Derived Coatings

**Figure 2:**
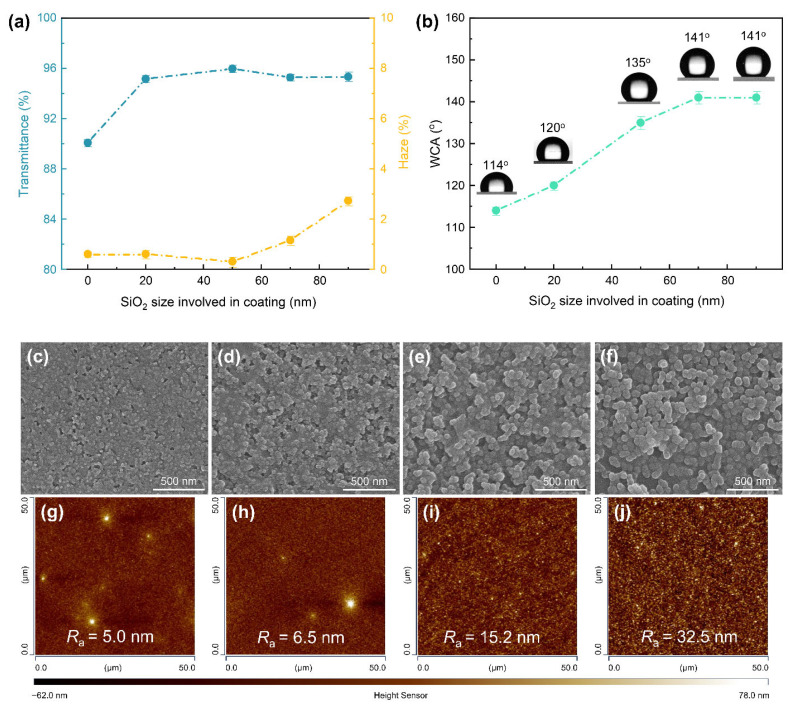
(**a**) Light transmittance and haze, and (**b**) WCA of the single-size NPs formed coatings. (**c**–**f**) SEM and (**g**–**j**) AFM images of the related coatings with particle size of 20, 50, 70 and 90 nm, respectively.

In the case of coatings derived from nano-size SiO_2_ NPs, Mie scattering is negligible in the visible region, and Rayleigh scattering could be the main cause of the increase of haze [27,44]. As shown in Figure 2a, coatings prepared with SiO_2_ NPs of 70 nm or smaller exhibited a haze below 1.5%. In this range, Rayleigh scattering was negligible relative to the substrate, meeting the requirements for most applications. While the transmittance was comparable, coatings with 90 nm NPs showed a haze 1.35 times higher than those with 70 nm NPs. This is in line with the theoretical ratio of their Rayleigh scattering intensity (I_90_/I_70_ = 2.45), demonstrating the dominant influence of Rayleigh scattering in haze value. The coating’s hydrophobicity (Figure 2b) showed a monotonic increase in WCA from 126° to 141° as particle size varied from 20 to 70 nm, but no further increase was observed with 90 nm NPs.

These size-dependent results can be attributed to the distinct surface morphologies of the coatings. As shown in the SEM and AFM images (Figure 2c,g), the coating derived from 20 nm NPs presents a smooth surface with few grooves and low roughness, due to the particles being fully embedded within the gel-like SHS network. With increasing particle size, a portion of the NPs becomes exposed, forming island-like agglomerates (Figure 2d,h). This leads to a moderate rise in surface roughness, which corresponds well with the observed increase in WCA. For coatings with 70 nm NPs, most particles are exposed, resulting in pronounced surface humps and grooves (Figure 2e,i). By contrast, increasing the size to 90 nm neither intensified these surface features (Figure 2f,j) nor led to a higher WCA. Additionally, bare glass showed an increase from 7° to 114° of WCA after passivation treatment with FAS-17, and a 1.90 of roughness factor was obtained according to the Wenzel contact model. These are close to the theoretically critical values of the flat surface (*θ*_0_ = 120°, *r* = 1.85) [45]. Therefore, in a single-particle-size system, surface roughness is primarily governed by the degree of NP exposure on the coating surface, which is a function of the initial particle size. Indeed, as reported in most literature [27,28,29,30,31], synchronous achievement of super-hydrophobicity (WCA > 150°) and ultra-low haze (H < 2%) for a transparent coating using single-size particles is not a simple process.

### 3.3. Optical Properties and Wettability of the Dual-Size Assembled Coatings

**Figure 3:**
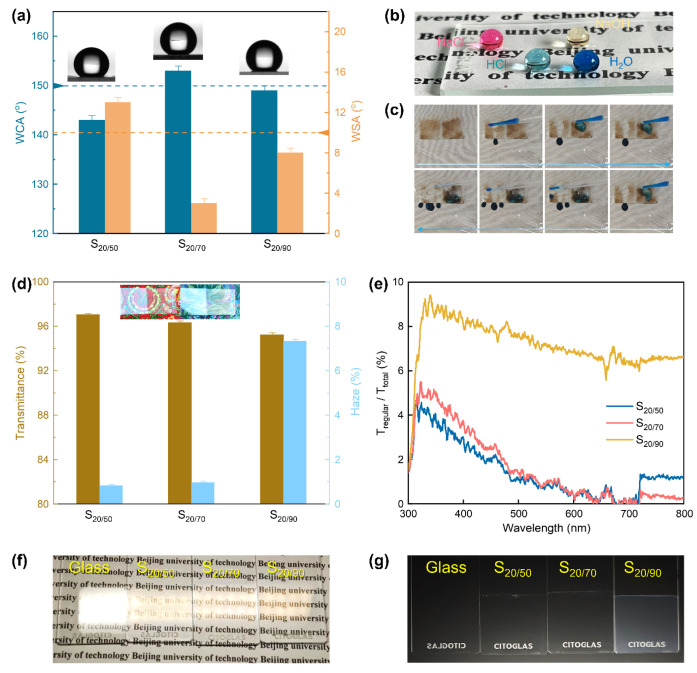
(**a**) WCA and WSA of the dual-size assembled coatings. Visual presentation of (**b**) hydrophobicity and (**c**) self-cleaning effect for S_20/70_ coating. (**d**) Light transmittance and haze, and (**e**) haze vs. incident wavelength of the dual-size assembled coatings. Visual presentation of (**f**) anti-reflection and (**g**) haze for dual-size assembled coatings.

Employing SiO_2_ NPs with a bimodal size distribution at an optimal ratio enabled the simultaneous optimization of hydrophobicity, optical transparency, and haze. In terms of hydrophobicity, all dual-size assembled coatings outperformed their single-size counterparts. Notably, the coating assembled from 20 and 70 nm NPs (S_20/70_) achieved the best performance, with a maximum WCA of 153° and a minimal WSA of 3° (Figure 3a). The ratio of dual-size NPs has a significant influence in hydrophobicity, and the optimal value emerges in a ratio from 2:3 to 3:2, while out of this region would cause WCA to drop below 150° (Figure S5). The superhydrophobic performance on the S_20/70_ surface is presented visually in Figure 3b. Specifically, deionized water, HCl solution, NaOH solution, and NaCl solution after dyeing present without exception near spherical water droplets on the surface. Figure 3c demonstrates the self-cleaning performance of the obtained superhydrophobic surface. To demonstrate the self-cleaning property, sand particles were dispersed on the coating. Rolling water droplets readily removed the contaminants, resulting in a surface that returned to its initial clean condition.

Regarding optical performance, the dual-size assembled coating demonstrates higher transmittance (Figure 3d), achieving a 5–7% increase compared to bare glass (90.1%). Notably, the transmittance is remarkably stable and shows little dependence on the size of the nanoparticles used. The transmittance decrease was minimal (under 2%) when the 50 nm NPs in S_20/50_ were replaced with either 70 nm (S_20/70_) or 90 nm NPs (S_20/90_). According to the principle of anti-reflection [46], coating thickness (d), incident wavelength (λ) and refractive index of the coating material (n1) should meet the relation d = λ/4n1. The thickness of silica (n1 = 1.43) coating under perfect optical interference is 70–136 nm in the visible region (400–780 nm). In fact, the coating thickness in our work was determined to be around 130 nm utilizing an AFM scan (Figure S6), staying within the ideal scope for anti-reflection. This is an important reason why single-size and dual-size derived coatings have similar transmittance. The well-marked anti-reflection performance of the coated glass is shown in Figure 3d inset and Figure 3f. Under fluorescent lights, the texts beneath are readily recognizable through the samples at an oblique viewing angle, owing to sensible suppression of visible light reflection. In contrast, bare glass shows a strong light reflection that completely screens the texts. Despite the prominent anti-reflection performance of all dual-size coatings, the haze of the S_20/90_ coating (7.3%) is significantly higher than that of the S_20/50_ and S_20/70_ samples (<1.0%). This increased haze reduces optical clarity, making the surface appear as if veiled in a thin mist (Figure 3e,g). Overall, the S_20/70_ coating successfully integrates super-hydrophobicity, effective anti-reflection, and extremely low haze, thereby achieving an optimal performance profile.

**Figure 4:**
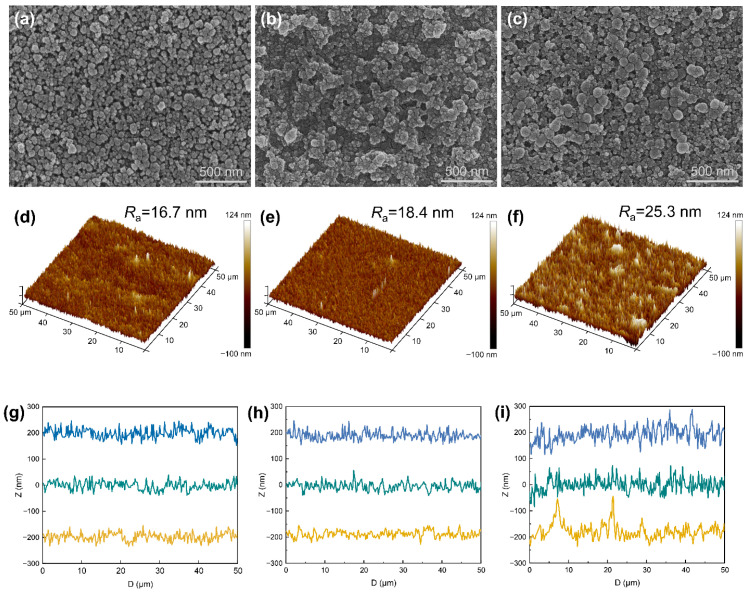
(**a**–**c**) SEM images, (**d**–**f**) 3D-AFM and (**g**–**i**) AFM scan profiles of S_20/50_, S_20/70_ and S_20/90_ samples, respectively.

To investigate the microstructure of the coatings and the influence of dual-size assembly on their properties, we characterized the samples using SEM and AFM. The observations demonstrate that the dual-size coatings possess a substructure notably distinct from single-size coatings, dependent on the specific particle compositions used. As seen in Figure 4a, the NPs almost separately disperse according to size when combining 20 and 50 nm-NPs. However, on replacement of the 50 nm NPs therein with larger NPs, such as 70 and 90 nm, the surfaces show pronounced particle aggregation, which creates a multi-scale rough structure consisting of humps and adjacent grooves (Figure 4b,c). Notably, S_20/70_ coating exhibits a significant sub-micro/nano-scale rough surface arising from the uniform distribution of moderate aggregates, demonstrating a texture-like profile (Figure 4b). By contrast, the surface particles of S_20/90_ coating display aggravated aggregations, so that form larger grooves and non-uniform structure form in micro-scale (Figure 4c). An AFM scan further illustrates the rough structure of the coatings (Figure 4d–f and Figure S7), in which the actual accumulation size on the surface was obtained by drawing a line and extracting the topography profile (Figure 4g–i). Among the three particle combinations, the S_20/70_ coating exhibited the best uniformity and the smallest micro-scale fluctuation, with an average roughness (Ra) of 18.4 nm. This result is highly consistent with the SEM observations. On the contrary, humps of a few micro-meters are widely spread in the S_20/90_ sample, and the corresponding Ra increased to 25.3 nm. For the S_20/50_ surface, despite the low roughness (Ra = 16.7 nm), non-uniform fluctuations in micro-scale are also clearly observed. The interesting variation of structure for dual-size assembled coatings can also be explained using the sphere dense packing model, where the particle sizes follow d/D = 0.414. For S_20/50_, the size ratio (0.4) is close to that of the ideal model, and this means dispersion among each other. For decreased size ratio such as S_20/70_ and S_20/90_, particles tend to agglomerate and accumulate. The smaller the size ratio, the more pronounced the particle aggregation during stacking.

Optical transparency, particularly the degree of haze, is largely governed by Rayleigh scattering resulting from surface roughness (Equation (2)). A uniformly flat surface tends to minimize Rayleigh scattering, thereby yielding low haze. However, according to the Cassie–Baxter relationship (Equation (1)), enhanced surface roughness contributes to a higher water contact angle. Consequently, it is challenging to achieve both low haze and super-hydrophobicity simultaneously. To resolve this dilemma, several earlier studies have suggested the possibility of incorporating abundant grooves and humps at the sub-micro-/nano-scale while maintaining an overall flat surface at larger scales [47,48]. Judging from the above results, S_20/50_ and S_20/90_ samples exhibit predominantly smooth and rough surfaces over large areas, respectively, leading to either low haze or high hydrophobicity. In the intervening gradation, the S_20/70_ sample possesses the combination of micro-scale uniformity and sub-micro/nano-size roughness, and thus nicely balances the haze–hydrophobicity competition. In this work, the key factor for the multi-scale rough structure is to control the size and distribution of aggregates on the substrate, which relies strongly on the involved particle gradation.

### 3.4. Effect of SHS on Properties of the Dual-Size Derived Coatings

**Figure 5:**
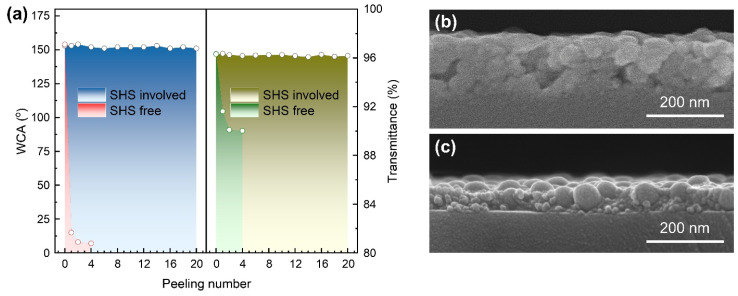
(**a**) Effect of tape peeling on transmittance and WCA for the S_20/70_ coatings with and without SHS. SEM cross sections for S_20/70_ coatings (**b**) with and (**c**) without SHS.

**Figure 6:**
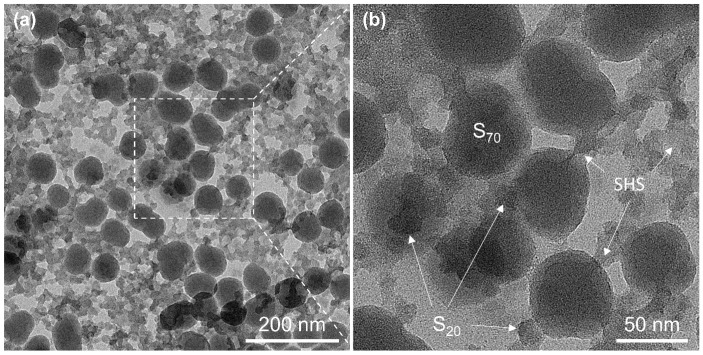
(**a**) Low-magnification and (**b**) high-magnification TEM images of S_20/70_–SHS composite sol.

**Figure 7:**
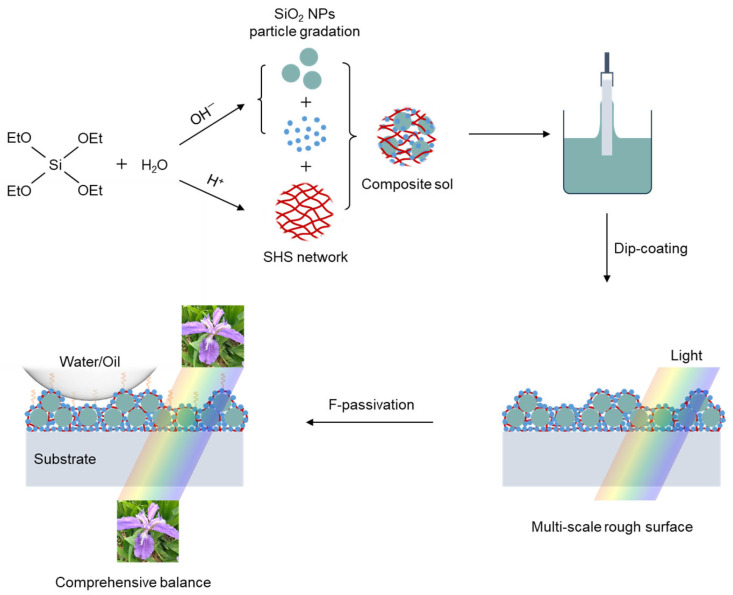
Sol-gel process for the production of dual-size assembled coating.

To reveal the role of SHS sol in construction of the robust transparent superhydrophobic coating, we compared the S_20/70_ coating with and without addition of SHS sol. As can be seen in Figure 5a, the just-prepared coatings hold similar features in terms of transparency and hydrophobicity. In the absence of SHS, the light transmittance reduced to the same level as that of bare glass after peeling twice with 3M tape. Nevertheless, in the SHS involved cases, the transmittance remained almost unchanged after 20 times peeling. In the meantime, the WCA value vs. peeling number shows the same trend as that of transmittance. Additionally, for the coating using only SHS, the deterioration of transmittance is barely detectable after tape peeling operations (Figure S8). This result indicates that SHS is essential to maintain high bonding strength between the coating and substrate. Under acid condition, SHS sol can be regarded as a partially hydrolyzed product, [Si(OC_2_H_5_)_4−x_(OH)_x_]_n_, which is apt to generate a cross-linking network between the silica clusters. When mixing dual-size SiO_2_ NPs with SHS sol, the NPs were wrapped and distributed by the interwoven three-dimensional network, possibly via further hydrolysis of SHS sol (Figure 6). The SEM cross-sectional image (Figure 5b) reveals that the 70 nm NPs are randomly dispersed within the coating layer. In contrast, the 20 nm NPs primarily fill the gaps between the larger ones. Furthermore, the interface layer adjacent to the substrate is composed solely of small NPs. This feature contributes to the coating’s performance by promoting a dense microstructure and maximizing the interfacial contact area with the substrate. In the absence of SHS, the SiO_2_ NPs formed a loosely packed structure with pronounced voids. This morphology indicates weak interparticle affinity as well as poor adhesion to the glass substrate. Thus, the presence of SHS promotes the uniform distribution of bimodal SiO_2_ NPs probably by hydrogen bonding, and generates tightly anchoring to the substrate at low temperature. Based on the above analysis, a schematic sol-gel process for the production of coating in this work was illustrated in Figure 7, demonstrating the stacking mode of the dual-size particles under the participation of SHS.

### 3.5. Robustness and Versatility of the Coatings

**Figure 8:**
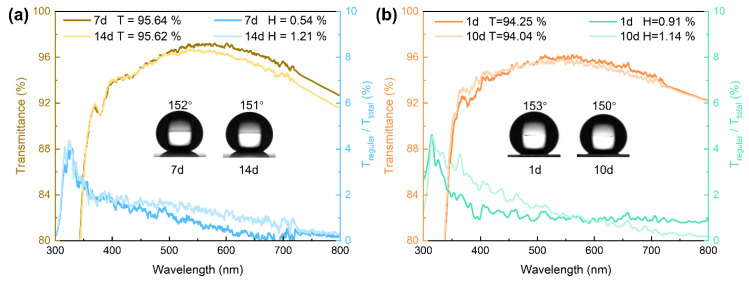
Light transmittance and haze curves after (**a**) ultra-low temperature and (**b**) hygrothermal tests for S_20/70_ coating.

To further assess the coating’s stability under extreme conditions, we performed tests involving extremely low temperatures and hygrothermal exposure. The sample was first cooled to −50 °C at a rate of 1 °C min^−1^, held at this temperature for two independent sets of 7 and 14 days, and then warmed back to room temperature at the same rate. A damp heat test lasting 1 and 10 days was performed under conditions of 60 °C and 95% relative humidity, following a similar procedure. Optical transparency and hydrophobicity were measured at each interval. The results are displayed in Figure 8. Notably, the coating exhibited excellent stability, maintaining its key parameters (95.64% transmittance, 1.21% haze, 151° WCA) without any significant loss after both a 14-day low-temperature test and a 10-day hygrothermal test. In addition, the coating has fine resistance to sunlight irradiation after exposure in an outdoor atmosphere for 6 months (Figure S9). These results demonstrate the coating’s remarkable environmental tolerance, indicating its strong potential for application in tropical, frigid, and other regions with large temperature and humidity variations.

**Figure 9:**
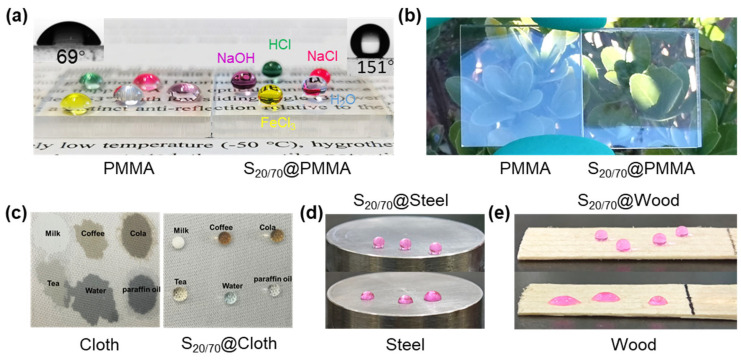
Visual presentation of highly transparent hydrophobic surface on (**a**,**b**) PMMA, (**c**) cloth, (**d**) steel and (**e**) wood.

To display the versatility of this dual-size assembled coating, we demonstrated the creation of a superhydrophobic transparent surface at low temperature on various substrates. When applying the optimal particle gradation to PMMA, i.e., pairing 20 nm NPs and 70 nm NPs, the surface became significantly hydrophobic (WCA = 151°) as clearly evidenced in Figure 9a, where the water, HCl, NaOH, FeCl_3_ and NaCl solution (1 M) droplets bead up, in stark contrast to the moderate hydrophily (WCA = 69°) of PMMA. In the meantime, transparency was maintained at a high value, with a transmittance of 95.31% and haze of 0.89%, respectively, showing a significant anti-reflection performance (Figure 9b). Benefitting from the flexible working procedure, this strategy can also create excellent hydrophobic/oleophobic surface for different substrates, such as nonwoven fabrics, stainless steel and pine wood (Figure 9c–e and Figure S10).

Overall, the silica coating developed here offers higher transparency than similar coatings reported previously [28,29,30,31], which represents a key advance. Although its water contact angle is slightly lower, this is compensated by the optical clarity achieved through precise particle size control—a strategy that reduces haze and increases transmittance. The low-temperature fabrication process further enhances its potential for large-scale use.

## 4. Conclusions

This study presents a systematic investigation into the construction of an anti-reflective, superhydrophobic surface via the rational assembly of dual-size silica particles. The main conclusions are as follows:

(1) A high optical transmittance (95.24%) with low haze (0.97%), high water contact angle (153°) and a low sliding angle (3°) were synchronously obtained for glass substrate by employing a sol-gel derived dual-size silica nanoparticles followed by dip-coating and passivation treatment.

(2) By controlling the hydrolysis of TEOS, a series of SiO_2_ sols with different average particle sizes can be synthesized. The S_20/70_ coating achieves an optimal balance between super-hydrophobicity and high transparency by virtue of its pronounced sub-micro-/nanoscale roughness.

(3) The transparent superhydrophobic silica coating can withstand extremely low temperature (−50 °C), a hydrothermal environment and long-term outdoor sunlight irradiation, which shows potential for applications in extreme environments.

(4) Transparent hydrophobic surface can be created on versatile substrates, including PMMA, cloth, steel and wood, using the low-temperature-processed, dual-size assembled silica coating.

## Data Availability

The original contributions presented in this study are included in the article. Further inquiries can be directed to the corresponding author.

## Supplementary Materials

The following supporting information can be downloaded at: https://www.mdpi.com/article/10.3390/ma18245601/s1, Figure S1: TEM images for the as-synthesized SiO_2_ sol with average size of (a) 20 nm, (b) 50 nm, (c) 70 nm and (d) 90 nm. Insets are the size distributions and digital pictures, respectively; Figure S2: TEM image of the as-synthesized SHS sol and the EDS elemental distributions; Figure S3: FTIR spectra for the as-synthesized SiO_2_ sol with average size of 20, 50, 70 and 90 nm; Figure S4: XPS survey of the coating before and after F-passivation; Figure S5: WCA of S20/70 sample with different particle gradations; Figure S6: 3D-AFM image and height scan for the S20/70 coating; Figure S7: 2D-AFM images of (a) S_20/50_, (b) S_20/70_ and (c) S_20/90_ coatings; Figure S8: Change of transmittance with tape peeling tests for the coating using just SHS sol; Figure S9: Pictures of S_20/70_ coating after six-month of outdoor exposure; Figure S10: (a) Visual presentation of hydrophobic surface on S_20/70_@cloth (b) WCA and OCA of S_20/70_@cloth before and after 30 min treatment of water-boiling, sonication, wash and soaking in 1 M of HCl aqueous solution, respectively.
