# Supplementary material for: Concertation of Anti-Reflective, Superhydrophobic Surface Based on Rational Assembly of Dual-Size Silica
Source: Materials (Basel). 2025 Dec 12;18(24):5601. doi: 10.3390/ma18245601 (PMC12734731; doi:10.3390/ma18245601)
Supplement: Supplementary file 1 [file materials-18-05601-s001.zip › materials-3996401-supplementary.pdf]

Supporting Information

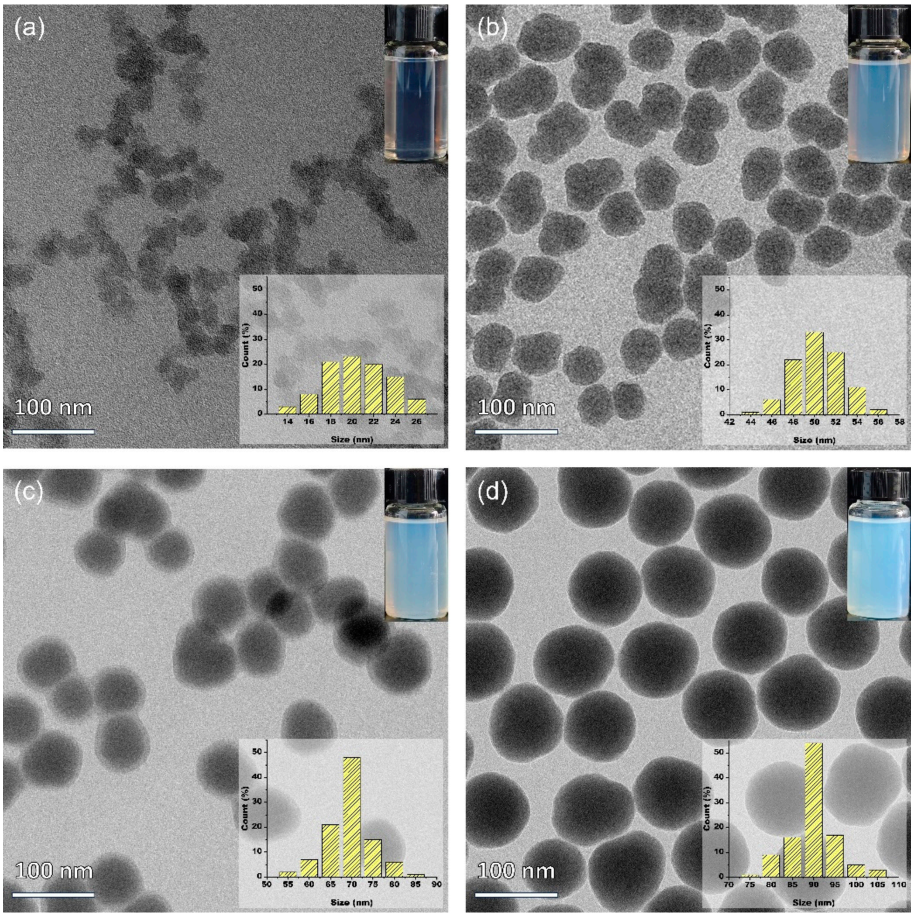

Figure S1. TEM images for the as-synthesized SiO_2 sol with average size of (**a**) 20 nm, (**b**) 50 nm, (**c**) 70 nm and (**d**) 90 nm. Insets are the size distributions and digital pictures, respectively.

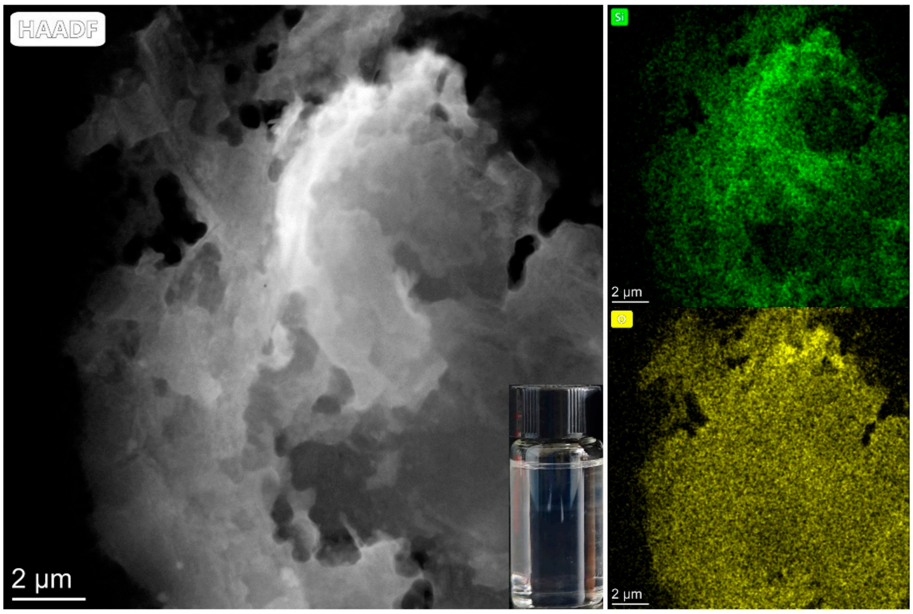

Figure S2. TEM image of the as-synthesized SHS sol and the EDS elemental distributions.

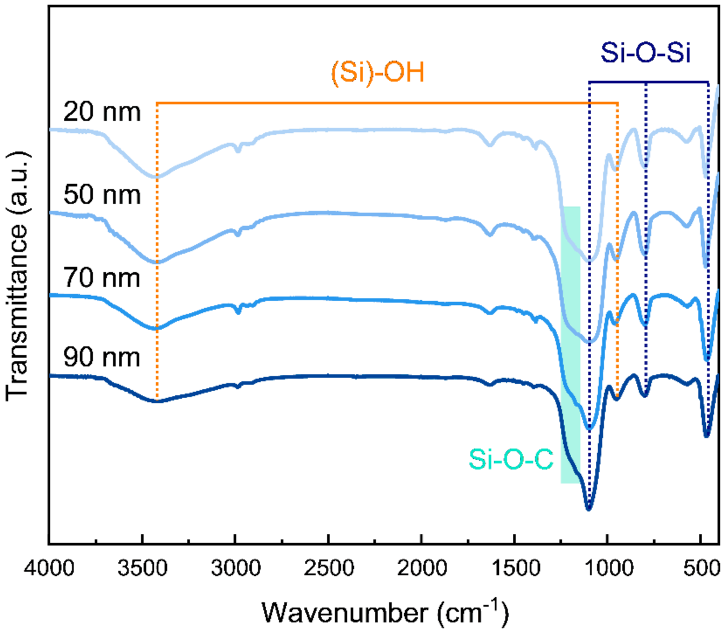

Figure S3. FTIR spectra for the as-synthesized SiO_2 sol with average size of 20, 50, 70 and 90 nm.

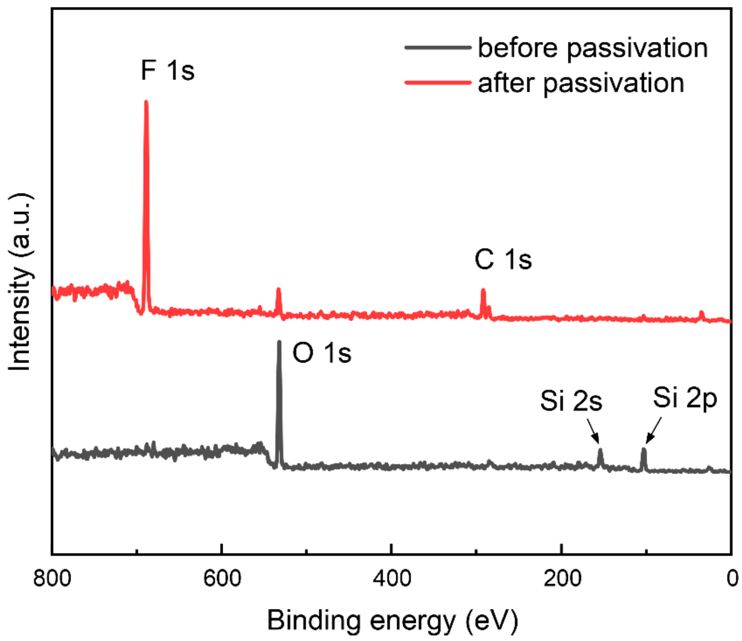

Figure S4. XPS survey of the coating before and after F-passivation.

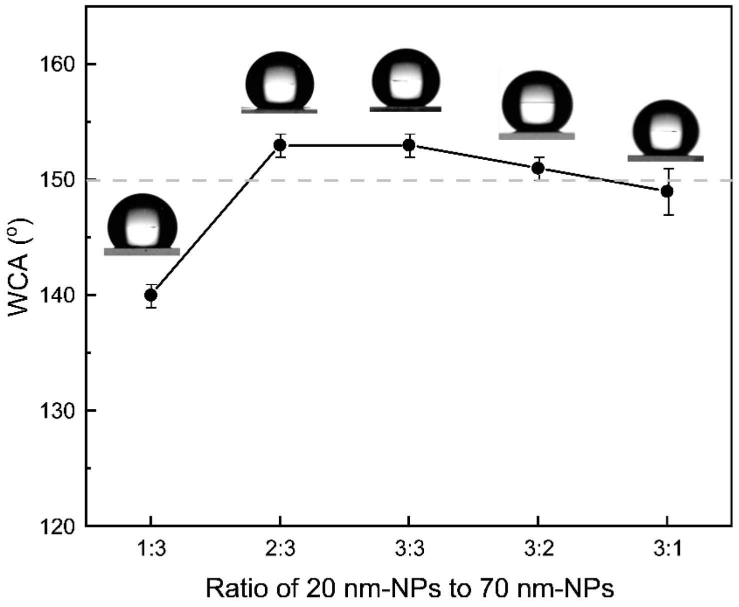

Figure S5. WCA of $S_{20/70}$ sample with different particle gradations.

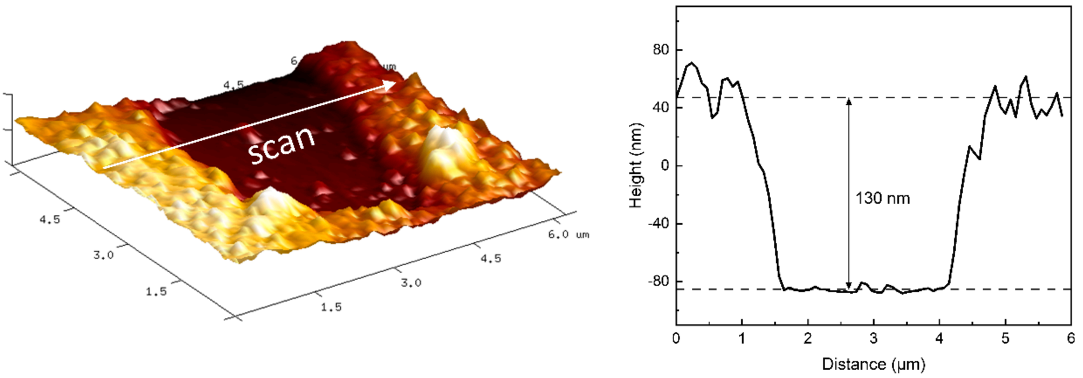

Figure S6. 3D-AFM image and height scan for the $S_{20/70}$ coating.

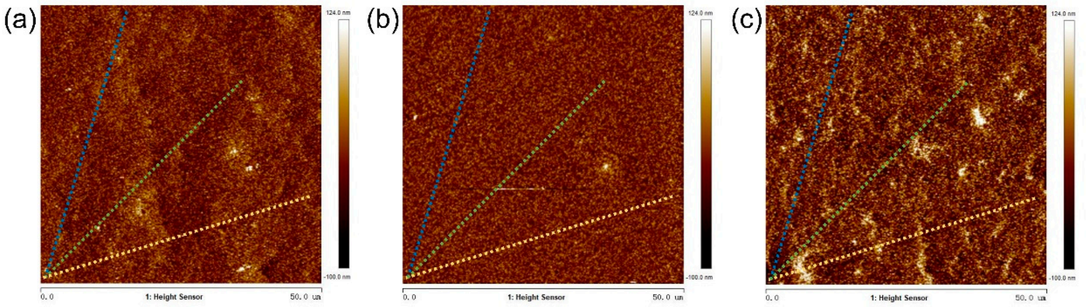

Figure S7. 2D-AFM images of (**a**) $S_{20/50}$, (**b**) $S_{20/70}$ and (**c**) $S_{20/90}$ coatings.

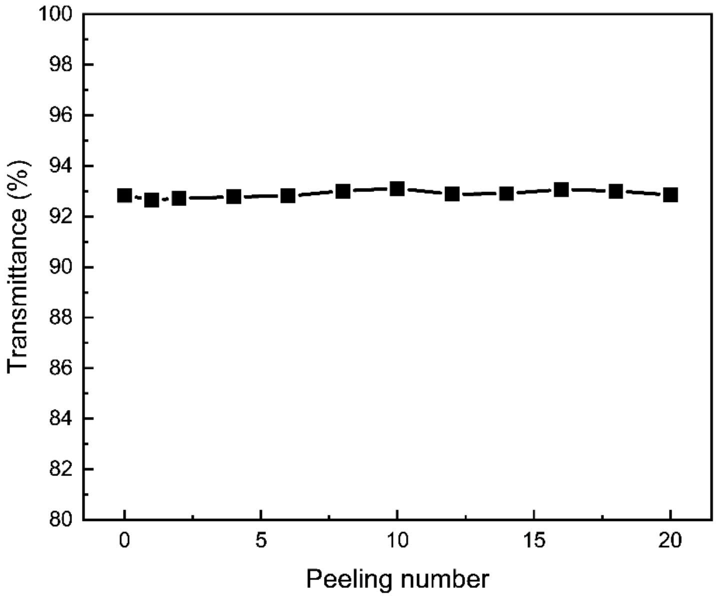

Figure S8. Change of transmittance with tape peeling tests for the coating using just SHS sol.

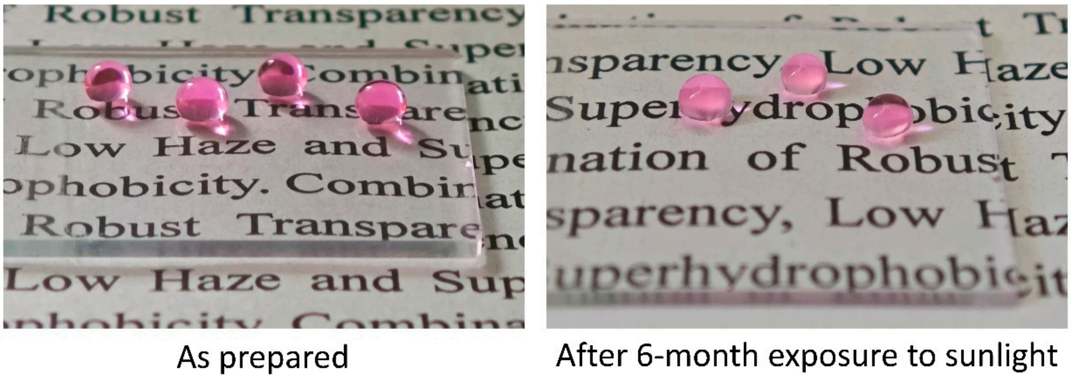

Figure S9. Pictures of $S_{20/70}$ coating after six-month of outdoor exposure.

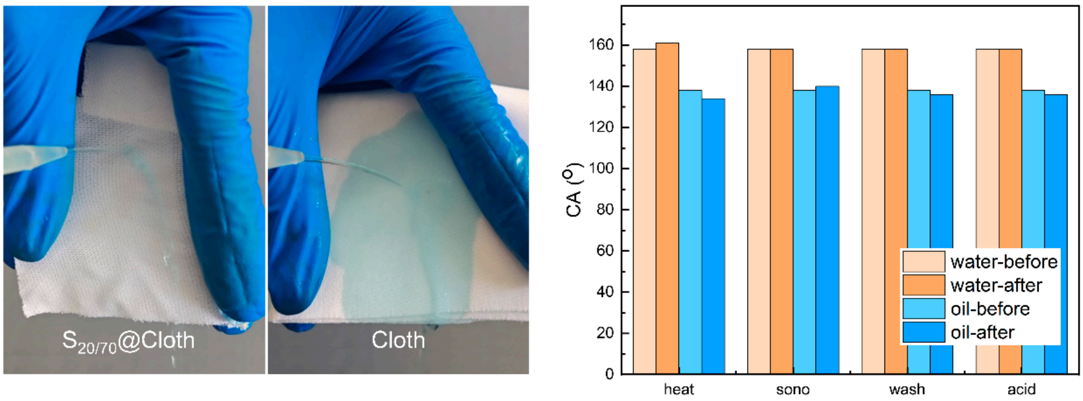

Figure S10. (**a**) Visual presentation of hydrophobic surface on $S_{20/70}$@cloth (**b**) WCA and OCA of $S_{20/70}$@cloth before and after 30 min treatment of water-boiling, sonication, wash and soaking in 1 M of HCl aqueous solution, respectively.
